# Spinal Cord Injury Causes Sustained Disruption of the Blood-Testis Barrier in the Rat

**DOI:** 10.1371/journal.pone.0016456

**Published:** 2011-01-26

**Authors:** Jennifer N. Dulin, Meredith L. Moore, Kevin W. Gates, Joanna H. Queen, Raymond J. Grill

**Affiliations:** 1 Department of Integrative Biology and Pharmacology, The University of Texas Medical School at Houston, Houston, Texas, United States of America; 2 Department of Neurosurgery, The University of Texas Medical School at Houston, Houston, Texas, United States of America; 3 Department of Integrative Biology and Pharmacology, The University of Texas Medical School at Houston, Houston, Texas, United States of America; Charité Universitaetsmedizin Berlin, Germany

## Abstract

There is a high incidence of infertility in males following traumatic spinal cord injury (SCI). Quality of semen is frequently poor in these patients, but the pathophysiological mechanism(s) causing this are not known. Blood-testis barrier (BTB) integrity following SCI has not previously been examined. The objective of this study was to characterize the effects of spinal contusion injury on the BTB in the rat. 63 adult, male Sprague Dawley rats received SCI (*n* = 28), laminectomy only (*n* = 7) or served as uninjured, age-matched controls (*n* = 28). Using dynamic contrast-enhanced magnetic resonance imaging (DCE-MRI), BTB permeability to the vascular contrast agent gadopentate dimeglumine (Gd) was assessed at either 72 hours-, or 10 months post-SCI. DCE-MRI data revealed that BTB permeability to Gd was greater than controls at both 72 h and 10 mo post-SCI. Histological evaluation of testis tissue showed increased BTB permeability to immunoglobulin G at both 72 hours- and 10 months post-SCI, compared to age-matched sham-operated and uninjured controls. Tight junctional integrity within the seminiferous epithelium was assessed; at 72 hours post-SCI, decreased expression of the tight junction protein occludin was observed. Presence of inflammation in the testes was also examined. High expression of the proinflammatory cytokine interleukin-1 beta was detected in testis tissue. CD68^+^ immune cell infiltrate and mast cells were also detected within the seminiferous epithelium of both acute and chronic SCI groups but not in controls. In addition, extensive germ cell apoptosis was observed at 72 h post-SCI. Based on these results, we conclude that SCI is followed by compromised BTB integrity by as early as 72 hours post-injury in rats and is accompanied by a substantial immune response within the testis. Furthermore, our results indicate that the BTB remains compromised and testis immune cell infiltration persists for months after the initial injury.

## Introduction

Men who have suffered traumatic spinal cord injuries (SCI) commonly become infertile, in large part because of a dramatic reduction in the quality of their seminal fluid. For these patients, sperm motility and viability are frequently poor [1]. Additionally, abnormalities of the semen are frequently exhibited after SCI; these include leukocytospermia, elevated levels of cytokines and reactive oxygen species within the seminal fluid, and the presence of seminal antisperm antibodies [2]–[4]. The decline in seminal quality after injury is rapid. In one report, sperm viability and motility were found to be as poor in men 16 days post-injury as that of men with chronic SCI [5]. It has been suggested that physiological and lifestyle factors such as scrotal temperature, bladder management, and ejaculation frequency might contribute to the poor quality of SCI patients' semen. To date, however, no definitive etiology has been identified [1].

The blood-testis barrier (BTB) is comprised of tight junctions between the Sertoli cells that line the seminiferous tubules of the testes; it divides the tubules into basal and adluminal compartments. The BTB has two important functions: (1) To maintain a specialized adluminal biochemical environment that is essential for the maturation of viable sperm, and (2) to confer immune privilege upon maturing germ cells [6]. Disruption of the BTB leads to the production of anti-sperm antibodies, causing immunological infertility [7]. Experimental BTB disruption in the rat has also been shown to cause reduction of tight junction protein expression, and germ cell loss from the seminiferous epithelium [8].

Recent studies indicate that pathological inflammatory processes after SCI are associated with damage of peripheral organs including the lung, kidneys, and bladder [9]–[11]. For example, damage to the bladder following SCI is characterized by a cellular inflammatory response, accompanied by decreased functional integrity of the uroepithelium due to a loss of tight junction proteins; this pathology persists for several months after SCI [10]. In the testes, it is known that systemic inflammation can induce damage and apoptosis in germ cells [12]. Additionally, it has been shown that proinflammatory cytokines disrupt the BTB and result in increased BTB permeability [13]–[15]. It is therefore conceivable that SCI-associated systemic inflammation might also adversely affect testicular function via breakdown of the BTB.

There is little published data on the effects of experimental SCI on the testes, and therefore it is not well-defined how variables such as the level of injury (e.g., cervical or thoracic) and injury model (e.g., transection, compression, contusion) may differentially affect testicular outcome. Huang and colleagues have made significant contributions toward understanding the effects of thoracic SCI on outcomes such as sperm damage and spermatogenesis in the rat [16]–[18]. In the current study, we have chosen to utilize a moderate, midline spinal contusion injury at thoracic level 10 (T10) with a 1 s dwell, using the rat as a model species. This “contusion/compression” injury model was selected for both its reproducibility and its close approximation of the blunt trauma injuries exhibited by a large percentage of patients in the clinic [19], [20]. Our laboratory utilizes a T10 injury model because of its common use by groups studying hindlimb function after SCI [19]. At this spinal cord level, there is no innervation to the testes; thus SCI at T10 does not directly alter neuronal input to testis tissue. Hence, the current study was designed to evaluate the effects of SCI on the testes in the absence of testicular denervation.

Since the effects of SCI on the BTB have not previously been reported, the overall aim of this study was to characterize BTB function in the rat following moderate spinal contusion injury. Here, we report functional alterations in the BTB in relation to histological changes in the testes in the acute and chronic phases of SCI.

## Materials and Methods

### Ethics Statement

We certify that all applicable institutional and governmental regulations concerning the ethical use of animals were followed during the course of this research. This study was carried out in strict accordance with the recommendations in the Guide for the Care and Use of Laboratory Animals of the National Institutes of Health. The protocol was approved by the Institute for Animal Care and Use Committee of The University of Texas Health Science Center (UTH NIH Assurance Number: A3413-01). All efforts were made to minimize suffering.

### Animal subjects and surgeries

A total of 63 adult, male Sprague-Dawley rats weighing 225–250 g were used in this study. All spinal cord injury surgeries were performed under anesthesia [ketamine (80 mg/kg), xylazine (10 mg/kg), acepromazine (0.75 mg/kg)] at a dose of 0.1 mL/100 g body weight. Spinal surgeries were performed using the Infinite Horizon spinal injury device (Precision Systems and Instrumentation, LLC, Fairfax Station, VA) [21]. Briefly, a laminectomy was performed at T10 and the vertebral column stabilized at T9 and T11. A moderate contusion injury was delivered using 150 kdynes of force with a 1 s dwell. Immediately following injury, the overlying muscles were sutured and the skin was closed with stainless steel wound clips. Sham-operated animals received a laminectomy without spinal cord contusion. Animals' bladders were manually expressed twice daily until control of bladder function was completely regained. Animals were treated twice daily for five days with buprenorphine (0.02 mg/kg). Animals received 0.9% saline *i.p.* twice daily for the first 72 h, as well as postoperative antibiotics (2.5 mg/kg Baytril) twice daily for up to 10 days to prevent infection.

Prior to DCE-MRI scans, animals were anesthetized with isoflurane (4%), intubated, and maintained under anesthesia (2–2.5% isoflurane, 30% oxygen, 67.5–68% air) by mechanical ventilation through a rodent ventilator for the entire duration of the scan (approximately 2 h). For intravenous delivery of the contrast agent, gadopentate dimeglumine (Gd) (287 mg/kg; Magnevist, Montville, NJ), the right jugular vein was cannulated, a vascular port with silicone tubing was implanted, and the incisions closed with suture. Animals used for DCE-MRI experiments were sacrificed either at 72 h post-SCI (*n* = 21), or at 10 mo post-SCI (*n* = 14). DCE-MRI scans were performed only once per animal. All animals were euthanized with beuthanasia (75 mg/kg) and transcardially perfused with ice-cold PBS. Testes were excised and immediately snap-frozen for histological analyses.

### DCE-MRI

To assess BTB permeability, dynamic contrast-enhanced magnetic resonance imaging (DCE-MRI) was performed. All DCE-MRI studies were performed on a 7-Tesla Bruker scanner (70/30 USR; Bruker Biospin, Karlsruhe, Germany). A 72-mm diameter birdcage volume coil was used for transmitter and receiver. Prior to each DCE-MRI scan, a quality assurance scan was performed to assess signal-to-noise ratio and magnetic field homogeneity. A 5-mm diameter cylindrical phantom was placed adjacent to the testes and used to monitor for any signal drop due to system instabilities in order to make corresponding compensation adjustments to the DCE-MRI signal during data analysis. Animals were oriented feet-first and supine on a Plexiglas bed. Respiratory rate and rectal temperature were monitored throughout the experiment with a physiologic monitoring unit. A pulse-oximeter was used to monitor heart rate and oxygen saturation levels. For the duration of the experiment, a heating system was used to maintain the body temperature at 33°C. This served to minimize temperature-responsive testes movement. The DCE-MRI scan consisted of a 2D T1-weighted multi-slice multi-echo scan to acquire contiguous axial images across the entire testes. Acquisition parameters were: repetition time [TR]  = 1200 ms, number of echo images  = 1, echo time [TE]  = 10.4 ms, field-of-view [FOV]  = 35 mm×43.75 mm, acquisition matrix  = 128×160, slice thickness  = 1 mm, number of slices  = 24, and number of repetitions  = 25. To avoid crosstalk between slices, a slice–interleaved acquisition scheme was used in addition to a gradient spoiler at the beginning of each slice acquisition and the use of Hermite RF pulses to improve the slice profile. The scan had a temporal resolution of 3 min, 12 s. Immediately following the third repetition, a ready-to-inject 0.2-mL bolus of Gd was administered into the jugular vein via the vascular port. Image intensities over a user-defined region of interest (ROI) encompassing only testes tissue were obtained using ParaVision 4.0 image analysis software. 8–10 integrated image intensities were obtained for each testis, and the mean image intensity for each group was calculated. The increase in signal intensity following Gd injection was expressed as a percentage change relative to baseline intensity, as previously described [22]. Data for right and left testes were analyzed separately in order to rule out the possibility of significant differences in permeability between the right- and left testes, but no significant differences were found (data not shown). Thus, the reported data reflects the sum total (right + left testes) of integrated image intensities for each animal.

### Immunofluorescent microscopy and immunohistochemistry

For all of the following histological procedures, frozen tissue was cryosectioned to a thickness of 10 µm, mounted to gelatinized slides, air dried, and fixed in −20°C methanol for 10 minutes. Every sixth section in series was chosen for histological analysis.

For immunofluorescent detection of endogenous immunoglobulin G (IgG), sections were blocked with 5% normal goat serum for 1 hr, then incubated with Alexa Fluor 488 anti-rat IgG (goat polyclonal, 1∶500, Invitrogen) overnight at 4°C on a rotating shaker. Sections were then stained with the nuclear stain DAPI (0.0007%) in PBS for 30 minutes, washed, dried, and coverslipped with Fluoromount-G (SouthernBiotech, Birmingham, AL). For all other immunofluorescent detection, sections were blocked first with 5% normal goat serum for 1 hr, then incubated with antibodies against occludin (rabbit polyclonal, 1∶250, Invitrogen), CD68 (mouse monoclonal, 1∶500, Serotec), or IL-1β (goat polyclonal, 1∶10, R&D Systems) overnight at 4°C on a rotating shaker. Tissue was washed and incubated with species-specific Alexa Fluor conjugated secondary antibodies (1∶500, Invitrogen) for 3 hr, stained with DAPI, washed again, dried, and coverslipped. Image stacks were generated using a Nikon A1R confocal microscope, and used to produce single, projected images. All efforts were made to maintain the same levels for laser power, gain, and contrast between image samples.

Cellular apoptosis was detected with terminal deoxynucleotidyl transferase-mediated dUTP nick end-labeling (TUNEL) staining, using the DeadEnd Colorimetric TUNEL System protocol (Promega). After fixation, tissue sections were permeabilized with Proteinase K. Tissue was then washed again in PBS and refixed in 4% paraformaldehyde. Tissue was washed again, equilibrated, and incubated with biotinylated nucleotide and recombinant Terminal Deoxynucleotidyl Transferase (rTDT) enzyme in a humidified chamber for 1 hr. After washes, sections were incubated in Streptavidin HRP solution for 30 min, and then developed using 3,3′ Diaminobenzidine as the chromogen. Tissue sections were counterstained with hematoxylin, washed, dried, and coverslipped. Mast cells were labeled by staining with toluidine blue. Sections were washed in PBS then dH_2_O, and stained with toluidine blue (0.1%) as previously described [23]. Sections were rinsed in dH_2_O, dried, and coverslipped. Light-level images were captured using an Olympus BX61 upright microscope with a SPOT Flex microscope digital camera.

### Immunoblot analysis

Animals used for immunoblot experiments (*n* = 28) were sacrificed and tissue was harvested as described above. Tissue was homogenized and total protein concentration was determined with the Pierce BCA protein assay (Thermo Scientific). Samples were resolved by SDS-PAGE, and transferred to PVDF membrane using a Trans-Blot SD semi-dry transfer cell (Bio-Rad). Non-specific antigens were blocked by incubation in Odyssey Blocking Buffer (LI-COR Biosciences, Lincoln, Nebraska) for 1 h at room temperature on a rotating shaker. Membranes were then incubated in primary antibodies against occludin (rabbit polyclonal, 1∶1000, Invitrogen) and the endogenous control marker β-actin (mouse monoclonal, 1∶15000, Abcam) at 4°C on a rotating shaker. Immunoreactivity was detected using species-specific IRDye infrared secondary antibodies (LI-COR Biosciences) and visualized with the Odyssey Infrared Imaging System (LI-COR Biosciences). Immunoreactivity was quantified using Odyssey software (LI-COR Biosciences).

### Statistical Analysis

Group data were analyzed by one-way analysis of variance, followed by a paired Student's t-test for comparisons between groups. Data are expressed as the mean ± standard deviation (s.d.), with *P* value <0.05 considered to be significant.

## Results

### DCE-MRI

**Figure 1:**
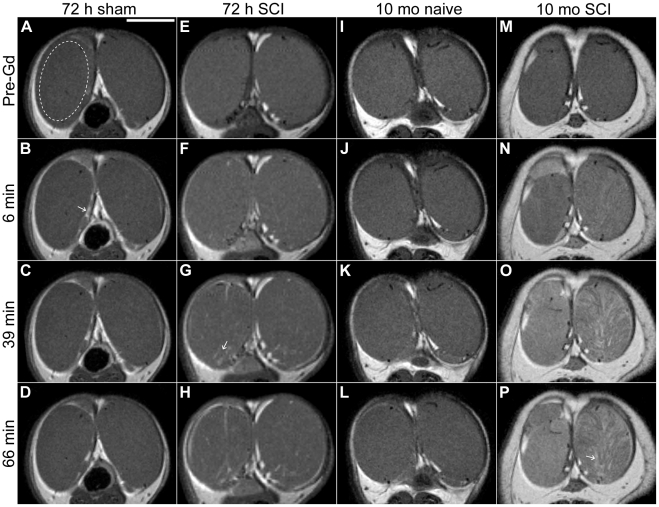
Representative dynamic contrast-enhanced MRI testes images. Each column displays an axial slice through the testes, as imaged at various time points during the course of the DCE-MRI scan. (Figure S1 provides a guide to interpreting testis anatomy in this figure.) Rows from top to bottom: before intravascular injection of the contrast agent gadopentate dimeglumine (Gd); 6 min-; 39 min-; and 66 min post-injection. Areas of Gd-induced contrast enhancement manifest as an increase in tissue brightness (for example, see Figure 1B, arrow). (A – D): Representative testes image from 72 h sham-operated group (*n* = 7). Dashed oval in (A) represents a typical user-defined region of interest that was used to calculate integrated image intensities. (E – H): Representative image from 72 h SCI group (*n* = 7). Arrow in (G) indicates a region of hyperintense tissue and increased BTB permeability. (I – L): Representative image from 10 mo uninjured (naïve) group (*n* = 7). (M – P): Representative image from 10 mo SCI group (*n* = 7). Arrow in (P) indicates a region of hyperintense tissue and increased BTB permeability. Scale bar  = 1 cm.

DCE-MRI is a noninvasive imaging technique that allows visualization of blood-organ barrier permeability using vascular contrast agents. The low molecular weight paramagnetic contrast agent Gd is administered *i.v.* and readily diffuses from the bloodstream to the extravascular space, but does not cross the BTB [22], [24]. DCE-MRI has previously been shown to be an effective indicator of BTB dysfunction in the rat and cat [22], [25]. To assess BTB function after SCI in rat, testis tissue permeability to Gd was examined using DCE-MRI at 72 h (acute) and 10 mo (chronic) following SCI. The 72-h time point was chosen for these experiments because it was shown to coincide with peak blood-spinal cord barrier permeability during the acute phase of SCI (unpublished data). (A guide to understanding testis anatomy on the MRI panels is provided in Figure S1.) Figure 1 shows representative DCE-MRI images of rat testes before and after vascular administration of the contrast agent Gd. Only regions of tissue within the tunica albuginea were analyzed (Figure 1A, dashed oval).

Testes of 72 h sham-operated animals (Figure 1, A–D) displayed a slight overall increase in signal intensity (SI) shortly after injection (Figure 1B), followed by a gradual reduction in SI over the course of the scan. 72 h age-matched, uninjured animals displayed contrast enhancement profiles similar to those of shams (data not shown). This slight global contrast enhancement in testes of non-SCI animals is attributable to Gd that diffuses into the interstitial space, but does not penetrate the BTB [22], [25]. By approximately one hour after administration of contrast agent, testes' SI still appeared slightly enhanced compared to pre-Gd baseline (Figure 1D). Testes of 72 h SCI animals (Figure 1, E–H) appeared similar to those of shams before Gd bolus (Figure 1E). However, contrast enhancement in 72 h SCI animals was exaggerated compared to shams; additionally, bright spots of enhancement were visible shortly after injection and gradually became more prominent over the course of the scan (Figure 1G, arrow). 10 month-old uninjured control animals displayed a contrast enhancement profile similar to that of 72 h shams (Figure 1, I–L). Conversely, testes of rats 10 mo after SCI displayed a rapid and substantial increase in SI from immediately after injection, and SI appeared to grow more enhanced over time (Figure 1, M–P). Contrast enhancement was not uniform throughout testis tissue, and distinct regions of especially high SI were often observed (Figure 1P, arrow).

**Figure 2:**
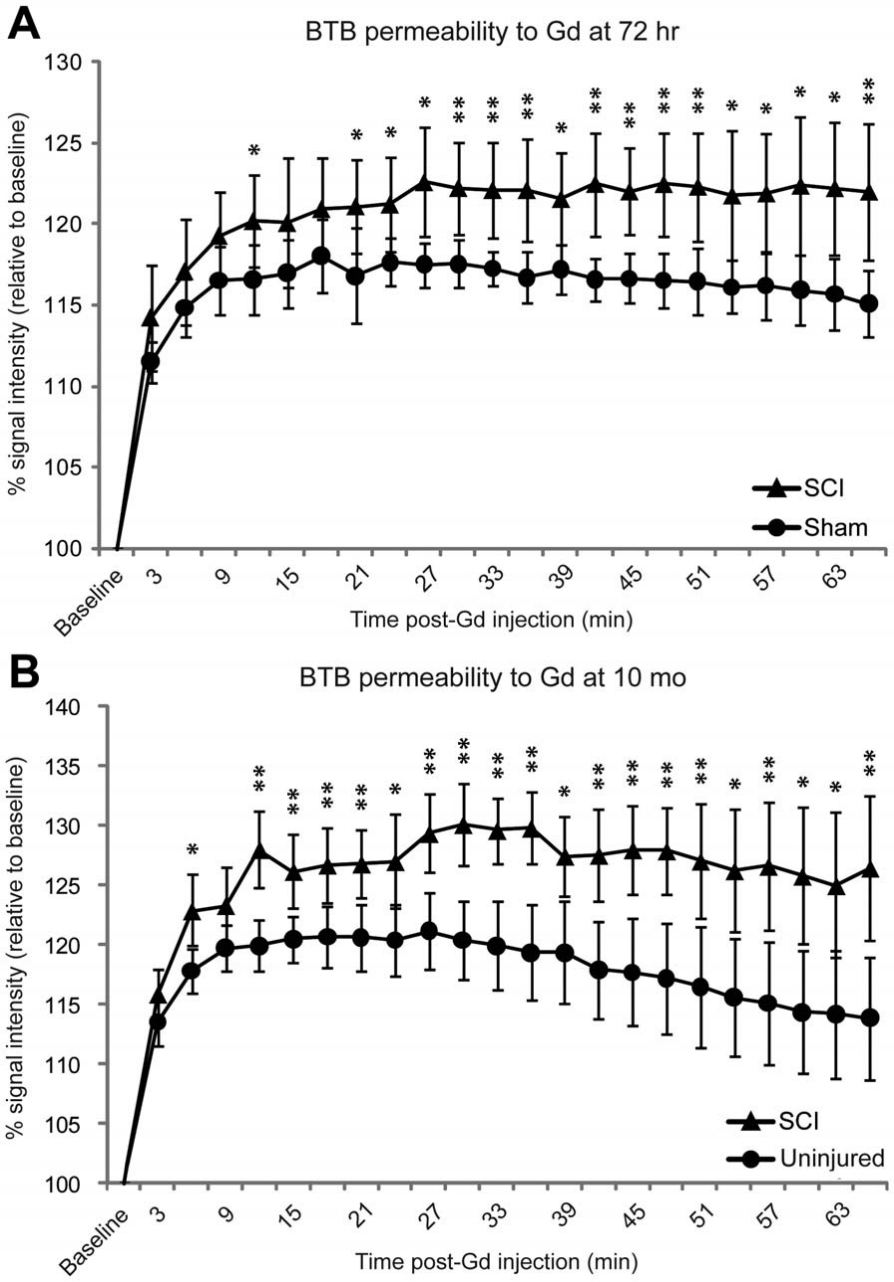
Blood-testis barrier permeability to Gd is increased in acute and chronic SCI. Gross BTB permeability is represented by integrated signal intensity (SI) of testes tissue after *i.v.* injection of gadopentate dimeglumine (Gd). All data are expressed relative to pre-Gd injection values (baseline). X-axis indicates number of min following Gd injection. Data points represent mean ± s.d. (A): Total SI for testes of 72 h SCI group (triangles, *n* = 7) compared to that of sham-operated group (circles, *n* = 7). SI was significantly higher in SCI group compared to shams by 12 min after Gd injection (*P* = 0.046), and remained significantly increased by the end of the scan (66 min, *P* = 0.009). (B): SI of 10 mo SCI testes (triangles, *n* = 7) compared to that of uninjured, age-matched control testes (circles, *n* = 7). SI of SCI group was significantly greater than that of controls by at 6 min after Gd injection (*P* = 0.011), and remained significantly increased at the end of the scan (66 min, *P* = 0.008). Asterisk (*) and double asterisk (**) indicate statistical significance *P*<0.05 and *P*<0.01, respectively.

In order to quantify BTB permeability, integrated image intensities of user-defined ROIs in testis tissue (Figure 1A, dashed oval) were obtained and plotted as a function of time relative to Gd injection (Figure 2). A significant increase in SI of the 72 h SCI group compared to shams (*P* = 0.046 at 12 minutes) was first observed early after *i.v.* administration of Gd (Figure 2A). For the remainder of the scan, SI was consistently higher in the SCI group compared to shams. In the 10 mo group, SI of the SCI group was also significantly higher than age-matched controls from early post-Gd injection (*P* = 0.011 at 6 minutes), and this trend was also sustained throughout the scan (Figure 2B). SI curves for the 10 mo uninjured group appeared to be slightly more variable among subjects compared to the 72 h sham group, possibly due to age-associated variability in BTB function.

### Histological evidence of BTB failure

**Figure 3:**
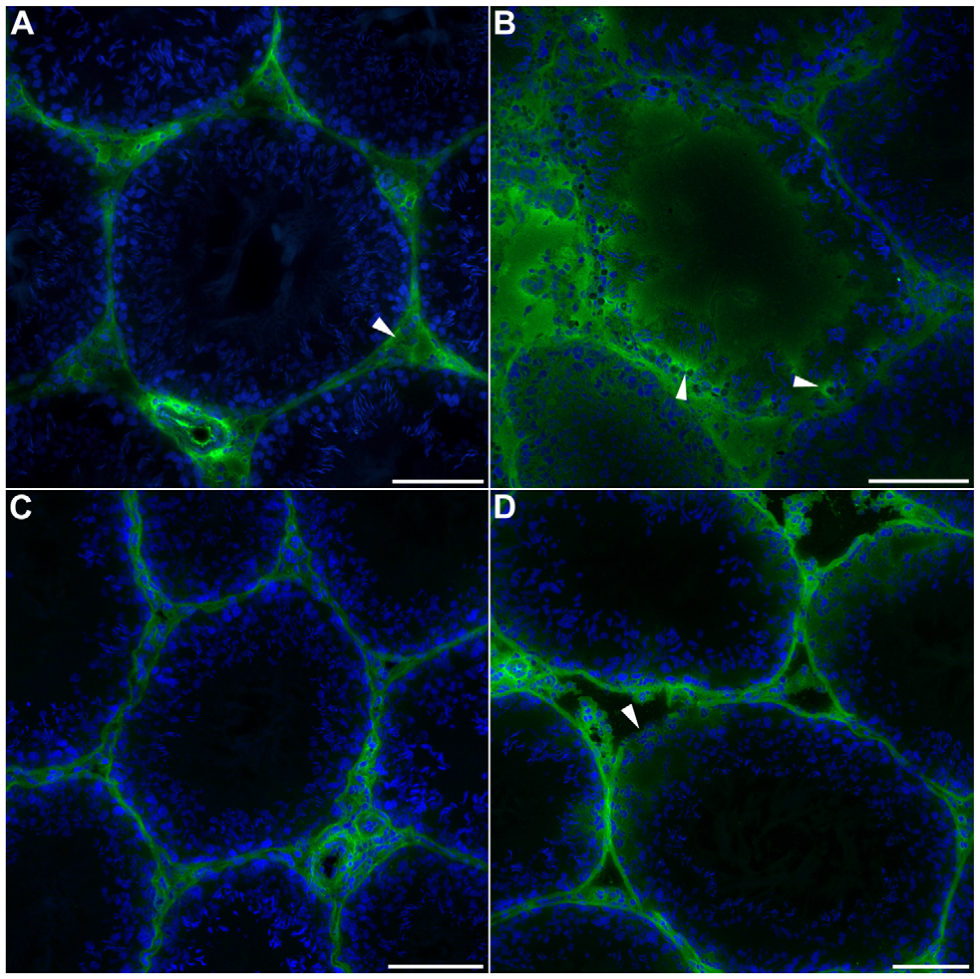
Seminiferous tubules are permeable to IgG following SCI. Immunofluorescent images of testis tissue labeled for the antibody protein immunoglobulin G (IgG; green) and counterstained for the nuclear stain DAPI (blue). (A): IgG was detected only within interstitial space of testes (arrowhead) in 72 h sham controls (*n* = 7). (B): IgG was detected within seminiferous tubules in testes 72 h after SCI (*n* = 7). Notably, some cells present within the seminiferous epithelium appeared to be closely associated with bright IgG immunoreactivity (arrowheads). (C): Seminiferous tubules of 10 mo age-matched, uninjured controls did not contain IgG (*n* = 7). (D): Diffuse IgG staining was present within seminiferous tubules of 10 mo SCI testes (*n* = 7). Additionally, the basal lamina of some tubules was partially degraded (arrowhead). Scale bars  = 100 µm.

To histologically examine BTB function, we examined testis distribution of immunoglobulin G (IgG), an abundant protein in the bloodstream that is normally restricted from the seminiferous tubules by the BTB (Figure 3) [26]. (Figure S2 is provided as an anatomical reference and guide to interpreting histological images.) In the 72 h sham group, IgG was detected only within the testis interstitial tissue (Figure 3A, arrowhead) and not within the tubules. In contrast, IgG deposits were frequently observed within seminiferous tubules 72 h after SCI (Figure 3B). In addition, strong IgG immunoreactivity surrounding some nucleated cells was detected within the seminiferous epithelium (Figure 3B, arrowheads). In 10 mo uninjured animals, IgG was only detected within the interstitial space (Figure 3C). However, diffuse IgG deposits are visible within the borders of seminiferous tubules in 10 mo SCI animals (Figure 3D). Moreover, the basal lamina appeared to be degraded in some IgG^+^ seminiferous tubules of 10 mo SCI testes (Figure 3D, arrowhead).

**Figure 4:**
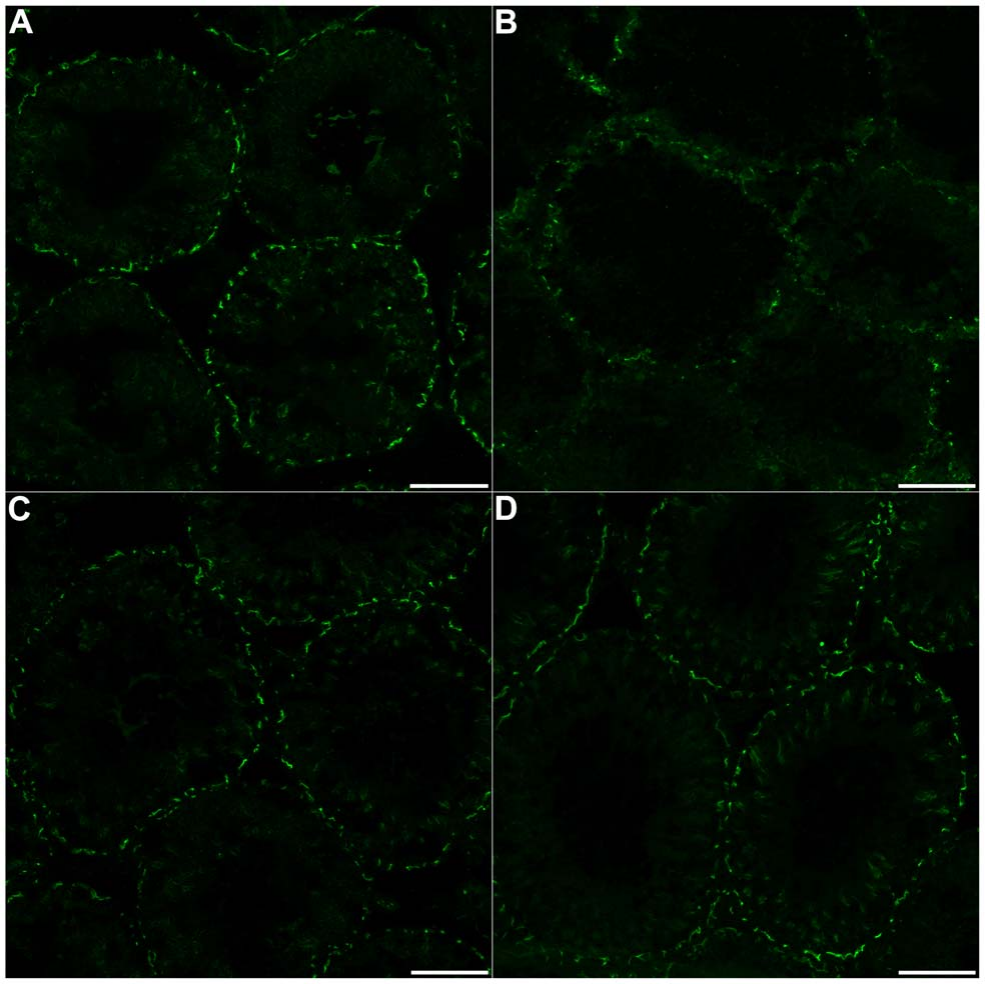
Occludin expression at the BTB is reduced during acute but not chronic SCI. Immunofluorescent images of seminiferous tubules labeled for the tight junction protein occludin (green). (A): Normal distribution of occludin at the BTB in 72 h sham-operated animals (*n* = 7). (B): 72 h after SCI, occludin immunoreactivity appeared reduced compared to sham controls (*n* = 7). (C): Occludin immunoreactivity in 10 mo uninjured animals appeared normal (*n* = 7). (D): Occludin distribution of 10 mo SCI group was similar to that of age-matched controls (*n* = 7). Scale bars  = 50 µm.

**Figure 5:**
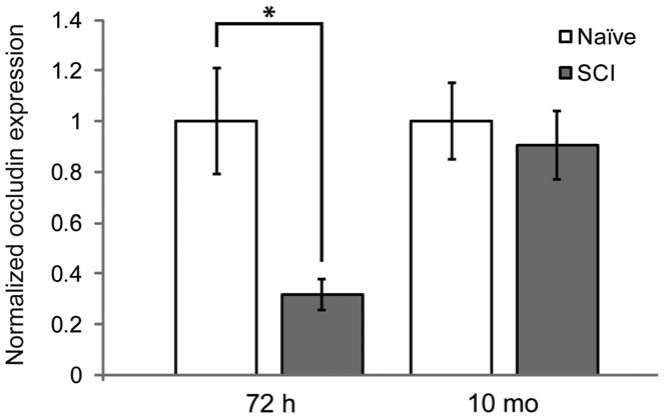
Testis occludin expression is decreased in acute SCI. Graph represents total testis occludin protein expression levels (mean ± s.d.) normalized to β-actin expression levels. Occludin expression was significantly decreased in 72 h SCI animals compared to naïve, uninjured controls [72 h naïve, 1.00±0.21 (*n* = 7); 72 h SCI: 0.32±0.06 (*n* = 7); *P* = 0.020 (asterisk)]. No significant difference was detected between expression levels of 10 mo SCI and 10 mo uninjured, age-matched control groups [10 mo naïve, 1.00±0.15 (*n* = 7); 10 mo SCI, 0.91±0.13 (*n* = 7); *P* = 0.282].

To investigate the structural integrity of the BTB, we examined distribution of the abundant BTB tight junction protein occludin in the seminiferous tubules (Figure 4). Occludin is located in tight junctions between Sertoli cells in the seminiferous epithelium, and normal occludin immunoreactivity appears as a regular, discontinuous pattern along the border of seminiferous tubules [27], [28]. In 72 h sham and 10 mo uninjured animals, occludin immunoreactivity at the BTB appeared normal (Figure 4A, C). However, 72 h after SCI, occludin at the BTB was dramatically altered (Figure 4B). Overall occludin levels were substantially decreased, and distribution patterns appeared abnormal in 72 h SCI testes. At 10 mo post-SCI, however, distribution of occludin at the BTB appeared normal (Figure 4D). Immunoblotting confirmed that total testes occludin protein levels were indeed significantly decreased at 72 h post-SCI compared to shams (*P* = 0.020), but no significant difference was detected between 10 mo groups (*P* = 0.282) (Figure 5).

### Germ cell apoptosis in acute SCI

**Figure 6:**
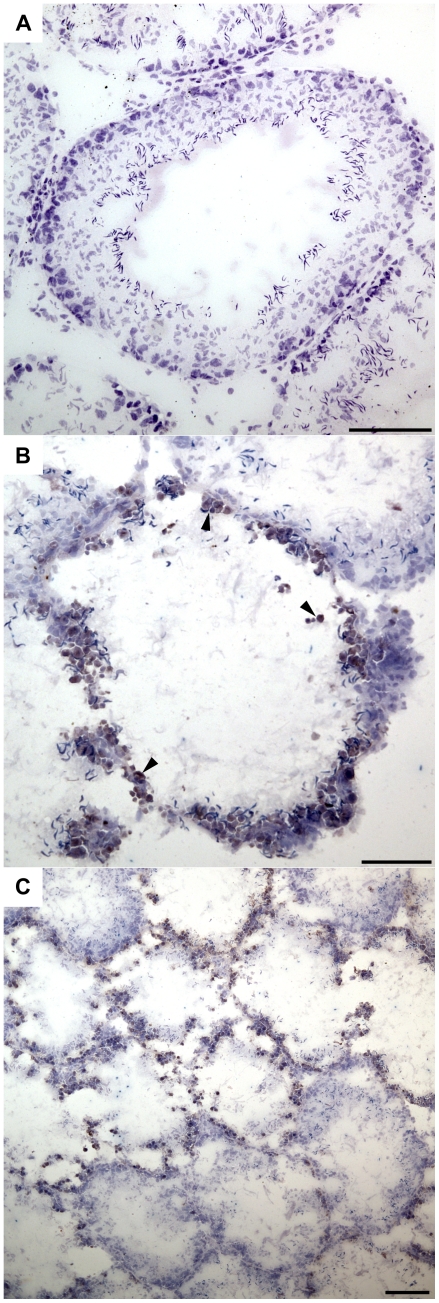
Apoptosis in the seminiferous epithelium during acute SCI. TUNEL staining revealed the presence of apoptotic cells (dark brown) in testis tissue. (A): Testes of 72 h sham-operated animals possessed very few detectable apoptotic cells (*n* = 7). (B): 72 h after SCI, seminiferous tubules possessed increased numbers of apoptotic cells (arrowheads) (*n* = 7). (C): Apoptosis was widespread in 72 h SCI testis. Note that seminiferous tubules in the SCI group contained varying amounts of apoptotic cells. Scale bars  = 100 µm.

Experimental disruption of the BTB has been shown to damage the seminiferous epithelium, inducing depletion and apoptosis of germ cells [8], [29]. To evaluate whether the observed BTB dysfunction following SCI was accompanied by germ cell death, TUNEL staining was carried out to detect apoptotic cells in the testis tissue. Though the seminiferous epithelium of sham controls possessed few or no cells undergoing apoptosis (Figure 6A), extensive cell death was detected within the tubules of 72 h SCI testes (Figure 6B). The localization of apoptotic cells within the tubules, as well as the number of apoptotic cells present, strongly suggested a germ cell identity. In addition, appearance of the apoptotic cells observed here was consistent with the appearance of apoptotic germ cells in the seminiferous epithelium in previously published reports [30], [31]. Finally, seminiferous tubules of 72 h SCI animals appeared to contain depleted numbers of germ cells. Thus, it is very likely that many of the cells undergoing apoptosis 72 h after SCI are germ cells. Widespread apoptosis was observed within many different seminiferous tubules, although the extent of apoptosis in each tubule varied (Figure 6C). Neither 10 mo uninjured controls nor 10 mo SCI animals exhibited evidence of germ cell apoptosis (data not shown).

### Immune cell infiltration following SCI

**Figure 7:**
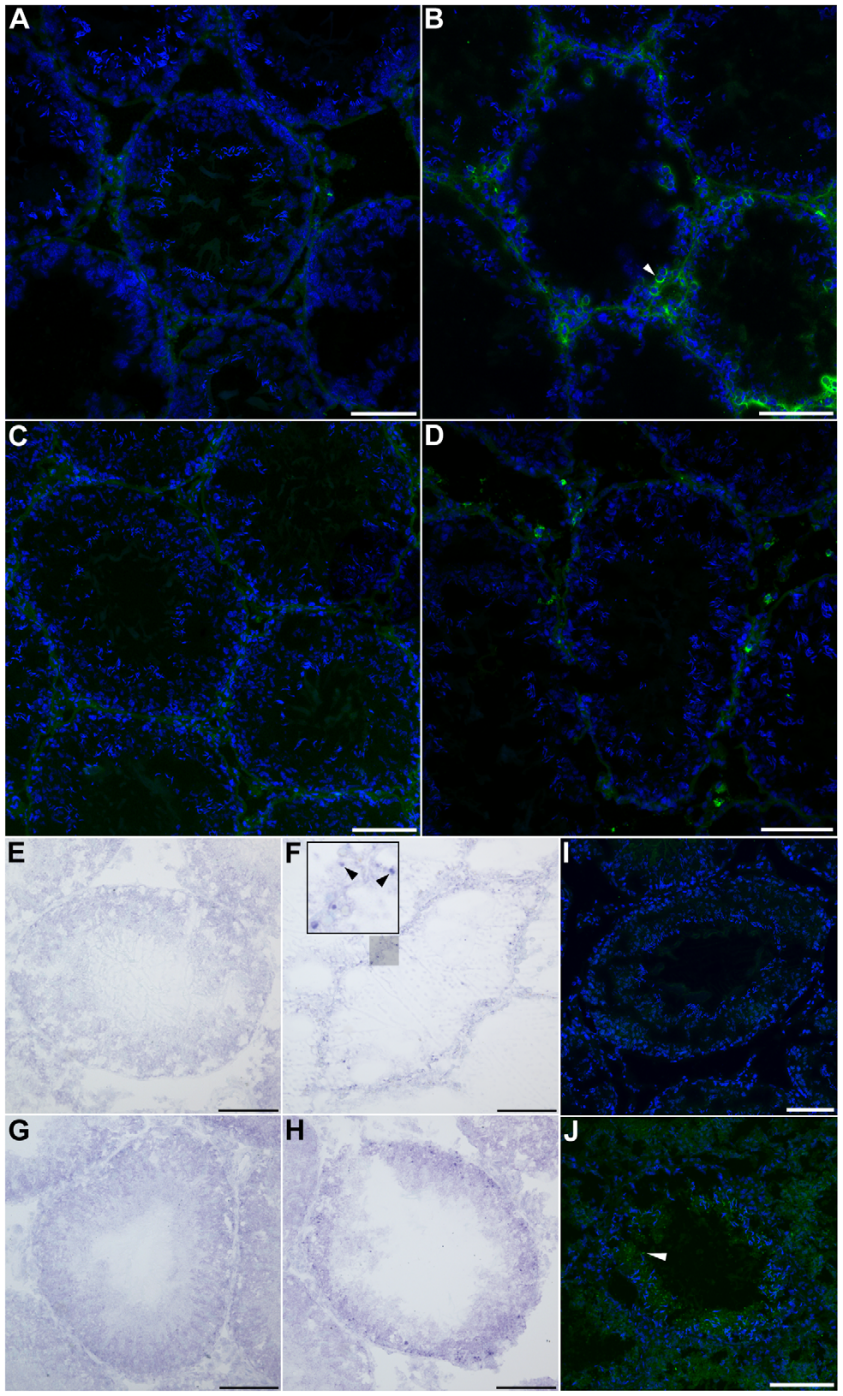
Immune cell infiltration in the testis tissue during acute and chronic SCI. (A – D): Immunofluorescent images of seminiferous tubules labeled for the macrophage antigen CD68 (green) and stained for the nuclear stain DAPI (blue). (A): Testes of 72 h sham-operated animals contained few CD68^+^ cells. (B): Up-regulation of CD68-expressing cells (arrowhead) within and surrounding the seminiferous tubules 72 h after SCI. (C): Few CD68+ cells were observed in testes of 10 mo uninjured controls. (D): 10 mo post-SCI, many CD68+ cells were detected surrounded the seminiferous tubules. (E – H): Light-level images of seminiferous tubules stained with toluidine blue to indicate mast cells (dark purple). (E): No detectable mast cells in or surrounding tubules in 72 h sham controls. (F): Mast cells (inset, arrowheads) were present within the seminiferous epithelium of testes 72 h post-SCI. Inset depicts a magnified view of the shaded region. (G): Few mast cells were present in testes of 10 mo uninjured controls. (H): Increased numbers of mast cells were detected within the seminiferous epithelium of testes 10 mo post-SCI. (I, J): Immunofluorescent images of 72 h testis tissue labeled for the pro-inflammatory cytokine interleukin-1 beta (IL-1β; green) and counterstained with DAPI (blue). (I): Testes of sham controls displayed no detectable IL-1β immunoreactivity. (J): 72 h after SCI, seminiferous tubules and interstitial testis tissue showed diffuse IL-1β immunoreactivity, and IL-1β^+^ cells were detected within the seminiferous epithelium. No IL-1β immunoreactivity was detected in testis tissue of the 10 mo groups (data not shown). Scale bars  = 50 µm.

Damage to peripheral organs following SCI has been shown to correlate with, and be mediated by, immune cell infiltration [9], [10]. In particular, testicular inflammation causes an influx of circulating monocytes and macrophages to the testes [32]. To investigate whether BTB breakdown following SCI is accompanied by the infiltration of these cells in the testes, immunostaining for the monocyte-macrophage marker CD68 was performed. Few CD68^+^ cells were observed in sham controls, and those observed were only located within the interstitial space (Figure 7A). At 72 h post-SCI, numerous CD68^+^ cells were detected within the interstitial space of the testes as well as within the seminiferous epithelium (Figure 7B). CD68^+^ cells were also present within the interstitial space of 10 mo SCI testes, but were not frequently observed within the seminiferous tubules (Figure 7D), whereas testes of 10 mo uninjured animals contained relatively few CD68^+^ cells and only within the interstitium (Figure 7C).

Mast cells play a key role in inflammation during the allergic response and defense against pathogens; they release molecules such as histamine that trigger the inflammatory response [33]. Mast cell presence within testis tissue and seminal fluid has been associated with infertility [34], [35]. Therefore, we investigated whether mast cells were present in testis tissue after SCI. Mast cells are identified by their distinct purple morphology upon toluidine blue staining (Figure 7F, arrowheads) [23]. Very few mast cells were detected within testes of 72 h sham animals (Figure 7E) and 10 mo naïve animals (Figure 7G). However, abundant numbers of mast cells were present within the seminiferous epithelium of both 72 h SCI animals (Figure 7F, arrowheads) and 10 mo SCI animals (Figure 7H).

The cytokine interleukin-1 beta (IL-1β) is a major proinflammatory mediator that promotes early inflammatory responses during pathological processes [36]. To investigate whether this pro-inflammatory signaling is present in the testes after SCI, IL-1β immunostaining was performed. Whereas testes of 72 h sham controls (Figure 7I) did not show IL-1β staining, 72 h SCI testes possessed diffuse immunoreactivity for IL-1β, both within the seminiferous tubules as well as in the interstitial space (Figure 7J). In addition, IL-1β^+^ cells were also detected within the seminiferous epithelium (Figure 7J, arrowhead). Neither 10-mo group displayed any IL-1β immunoreactivity (data not shown).

## Discussion

Here, we have described a considerable decline in BTB integrity subsequent to moderate spinal contusion injury in the adult rat. We show that normal BTB function—the exclusion of blood-born substances from the seminiferous tubules—is considerably impaired from as early as 72 h after SCI. Furthermore, our MRI data suggests long-term deficits in BTB function are sustained as late as 10 mo after SCI. We also demonstrate testis immune cell infiltration at both acute and chronic SCI. Lastly, we provide evidence that early post-SCI, the environment within the testis is proinflammatory, BTB structural integrity is compromised, and there is extensive apoptosis and depletion of germ cells.

DCE-MRI is a technique widely used to study permeability of blood-organ barriers. The vascular contrast agent Gd does not penetrate the intact BTB, and as such DCE-MRI has been demonstrated to be an effective measure of BTB permeability *in vivo* [22], [25]. We now report contrast enhancement profiles following SCI in the rat that indicate a substantially dysfunctional BTB. To ascertain whether these observed effects could be attributed to the invasive surgical procedures performed in this study, or to the direct effect of injury to the spinal cord, we compared the contrast enhancement profiles of 72 h sham-operated animals to 72 h naïve controls. We did not detect any significant differences, either qualitatively (by objective analysis of MRI images) or quantitatively (by comparison of SI curves) in the BTB permeability of shams versus naïve controls (data not shown). From this, we conclude that the observed increases in BTB permeability are attributable to SCI alone, and we also propose that the use of 10 mo age-matched, naïve controls is an appropriate control for this study. However, we cannot discount the possibility that the effects of sham-surgery may alter properties of the BTB over prolonged periods of time. This possibility will be addressed in future investigations of the chronic effects of SCI on the BTB.

The antibody protein IgG, an abundant component of the bloodstream, is restricted from penetrating the intact BTB [26]. Deposits of immunoglobulins have frequently been detected within the seminiferous tubules of rats immunized with testicular antigens or treated with adjuvants to disrupt the BTB [37], [38]. Here, we report the presence of IgG deposits within rat seminiferous tubules at 72 h following SCI. Together with our DCE-MRI results, this provides compelling evidence that the BTB is compromised during the acute phase of SCI. IgG deposits were also observed, though to a much lesser extent, within seminiferous tubules of the 10 month post-SCI rat. While this may indicate a chronic loss of BTB integrity, we do not discount the possibility that these IgG deposits are actually the uncleared remains of IgG deposited early after injury. It is also conceivable that during chronic SCI, the BTB is differentially permeable to smaller molecules such as Gd (0.9 kD), and relatively impermeable to larger molecules such as IgG (150 kD). We plan to assess the seminiferous tubule permeability of exogenously added, fluorescently-tagged markers of various molecular weights in future chronic SCI studies.

Based on our results, it is likely that decreased expression of tight junction proteins such as occludin contribute to BTB dysfunction during the acute phase of SCI. Multiple other protein components of the BTB are critical for normal barrier opening and closing during spermatogenesis [39]. It is likely that other BTB proteins, while not examined in the current study, play early or sustained roles in ongoing BTB dysfunction. Recently, it was shown that SCI disrupts tight junctions within the uroepithelium of the bladder, causing uroepithelial dysfunction from the acute to chronic phases of injury [10]. In addition, there is evidence of a persistent inflammatory process that promotes elevated blood-spinal cord barrier permeability, and persists during the chronic phase of SCI [40]. Organ dysfunction and failure is common in SCI patients [41]. We therefore speculate that a sustained systemic response after SCI might impact multiple peripheral tissues and organ systems.

Here, we have reported the presence of IL-1β in testis tissue during the acute phase of SCI. IL-1β is a potent proinflammatory cytokine secreted by macrophages, and its presence is strongly indicative of the acute phase inflammatory response of host defense in disease. It promotes activation of immune cells such as macrophages and neutrophils, causing them to release tissue-damaging free radicals and proinflammatory peptides, and phagocytose invading pathogens [42]. Thus, presence of IL-1β within the testes 72 h after SCI is indicative of a pathological, proinflammatory process that occurs early following injury. The specific effects of IL-1β signaling following SCI are not clear. It was previously shown that IL-1β expression is also up-regulated in a rodent model of experimental testicular ischemia-reperfusion. In this study, IL-1β was sufficient to activate testicular endothelial signaling pathways promoting neutrophil recruitment, and causing germ cell-specific apoptosis [43]. Our results indicate that IL-1β secretion is correlated with extensive germ cell apoptosis at 72 h after SCI in the testes (Figures 6, 7). IL-1β is also known to affect integrity of tight junctions; for example, IL-1β increases the permeability of tight junctions between epithelial cells lining the intestines, and vascular endothelial cells at the blood-brain barrier and blood-retinal barrier [44]–[46]. Proinflammatory cytokines are known to play a role in BTB dynamics as well [13], [14], [47]. It is therefore plausible that IL-1β signaling early after SCI is implicated in BTB opening. However, as there is little data describing the role of this cytokine in the testes, future studies are necessary to understand its role.

Breakdown of the BTB exposes germ cells to the immune system, enabling systemic autoimmunity to develop [48], [49]. Anti-sperm antibodies have been found in the blood and seminal fluid of men with SCI, and sperm autoimmunity has been acknowledged as an important contributing factor to SCI-related male infertility [50]–[52]. However, sperm autoimmunity in men who have received a SCI has not previously been associated with BTB dysfunction. Here, we have demonstrated substantial infiltration of immune cells into the testes, showing both an early immune response during acute SCI, and a sustained presence of immune infiltrate in chronic SCI. Interestingly, this histopathology is similar to that of experimental autoimmune orchitis (EAO), an animal model of sperm autoimmunity induced by immunization with sperm or testicular antigens. EAO pathology—chiefly, immune cell infiltration into the seminiferous epithelium—is believed to be associated with BTB dysfunction and male infertility [48], [49], [53]. We posit that compromised BTB integrity following SCI may lead to infiltration of immune cells, as in EAO. It is possible that this immune infiltrate may play a direct role in the early apoptosis of germ cells we have described. Lysiak *et al.* have demonstrated that infiltration of neutrophils is essential for ischemia-induced germ cell-specific apoptosis in the mouse [54]. There is much evidence that immune cell infiltration to the testes is associated with reproductive deficits in men. Mast cells in testis tissue and seminal plasma are associated with infertility [34], [35]. Additionally, increased numbers of mast cells and CD68^+^ macrophages in the testes are associated with abnormal spermatogenesis [55]. CD68^+^ macrophages in testis tissue are mostly proinflammatory [56]; furthermore, proinflammatory cytokines are involved in testis tissue damage, germ cell damage and apoptosis, and BTB disruption [13], [15], [47], [53], [57], [58]. Therefore, it is likely that inflammation in the testes is closely associated with the germ cell apoptosis we have observed at 72 h following SCI.

The mechanism underlying male infertility after SCI is not currently understood. Presently, only a few groups have investigated this question in an experimental model. Huang and colleagues have published multiple studies describing the mechanisms by which SCI leads to defects in spermatogenesis. Chow et al. have speculated that testicular dysfunction during chronic SCI might result from deficient neural input to the testes [59]. A similar phenomenon has been observed in the bladder; it was shown that SCI-induced bladder dysfunction could be attenuated by silencing the neural input into the bladder [11]. In the rat and human, the testes are innervated by the genitofemoral nerve which originates at L1-2, thus testicular deficits arising after injury to the cord at T10 are not likely attributed to testicular denervation [60]. However, it is conceivable that SCI-associated deregulation of neural activity arising from the L1-2 spinal segments might affect testicular function. Further examination of this possibility would require a detailed study exploring the effects of neural inhibition of that input either before or immediately after SCI. Compelling evidence against the argument for altered testicular neural input comes from a study in which Brackett et al. showed that a group of men with SCI at levels above L1 (cervical, *n* = 59; T1–T6, *n* = 39; T7–T12, *n* = 42) possessed double the amount of dead, immotile sperm compared to normal men, regardless of the level of injury [61]. These men showed no difference, by level of injury, in the mean amount of dead sperm within their seminal fluid. Though a similar study has not yet been performed in the rat, these results lend support to the theory of a systemic pathological mechanism, rather than altered neural input, that induces testicular deficits following SCI.

Here we have described, using non-invasive imaging and immunohistological methods, a breakdown in BTB function following SCI in the adult rat. Our results provide evidence that an injury-induced mechanism causing BTB compromise is present early after SCI, and MRI data indicates that small-molecule BTB permeability may be sustained indefinitely. We have also identified histological evidence of an immune response in the testes 72 h subsequent to SCI, which is also exhibited as late as 10 months after injury. Together, these results suggest that there exists a loss of BTB integrity which may persist throughout the lifetime of the subject. This study both describes a novel effect of SCI on a peripheral blood-organ barrier, and provides new insight into the pathophysiology of infertility in men with SCI.

## Supporting Information

Figure S1 **Labeled anatomical guide to interpreting rat testes MRI.** (A): Cartoon outline of a rat in the supine position, as during MRI scanning. Red line through testes indicates the position of a representative image slice in Figure 1. (B): Cartoon depicting the cross-sectional (axial) view of the slice in (A). This image corresponds to the actual MRI slice shown in (C). Legend: 1  =  right testis; 2 =  left testis; 3 =  epididymis; 4 =  tunica albuginea; 5 =  scrotum; 6 =  tail. (C): Actual MRI image of testes from a sham-operated animal.
(TIF)

Figure S2 **Anatomical guide to the testes and seminiferous tubules.** (A): Cartoon outline of a rat illustrating the approximate size, shape, and location of testes. (B): Cartoon cross-section of a rat testis. Legend: 1 =  collecting duct; 2 =  epididymis; 3 =  seminiferous tubules. (C): Photograph of a testis from a naïve rat that received intravascular Evans Blue (an albumin-binding dye). The blood vessels (1) on the testicular surface appear blue because they contain albumin-rich blood. Red line indicates plane of sectioning for histological studies. Inset shows the coiled seminiferous tubules (2), visible in contrast against the darker blue interstitial space (3). Ruler denotes length in cm. (D): Cartoon outline of a representative histological section of testis tissue. Legend: 1 =  seminiferous tubule; 2 =  interstitial space; 3 =  blood vessels. (E): Actual immunofluorescent image of the seminiferous tubule depicted in (D). The nuclear stain DAPI is shown in blue. Asterisk indicates the lumen of a seminiferous tubule. Immunoreactivity for the blood-born protein immunoglobulin G is shown in green, to emphasize the distinct border between the interstitial space and the seminiferous tubules. Red box corresponds to (F). (F): Cartoon depicting cells of the seminiferous epithelium and the blood-testis barrier. Legend: 1 =  Sertoli cell; 2 =  tight junction (blood-testis barrier); 3 =  basal lamina; 4 =  spermatogonium; 5 =  spermatocyte; 6 =  spermatid; 7 =  interstitial space. The blood-testis barrier separates the seminiferous tubule into two distinct compartments: basal (yellow) and adluminal (orange).
(TIF)
