# Supplementary figures and images for: Spinal Cord Injury Causes Sustained Disruption of the Blood-Testis Barrier in the Rat
Source: PLoS One. 2011 Jan 26;6(1):e16456. doi: 10.1371/journal.pone.0016456 (PMC3027675; doi:10.1371/journal.pone.0016456)

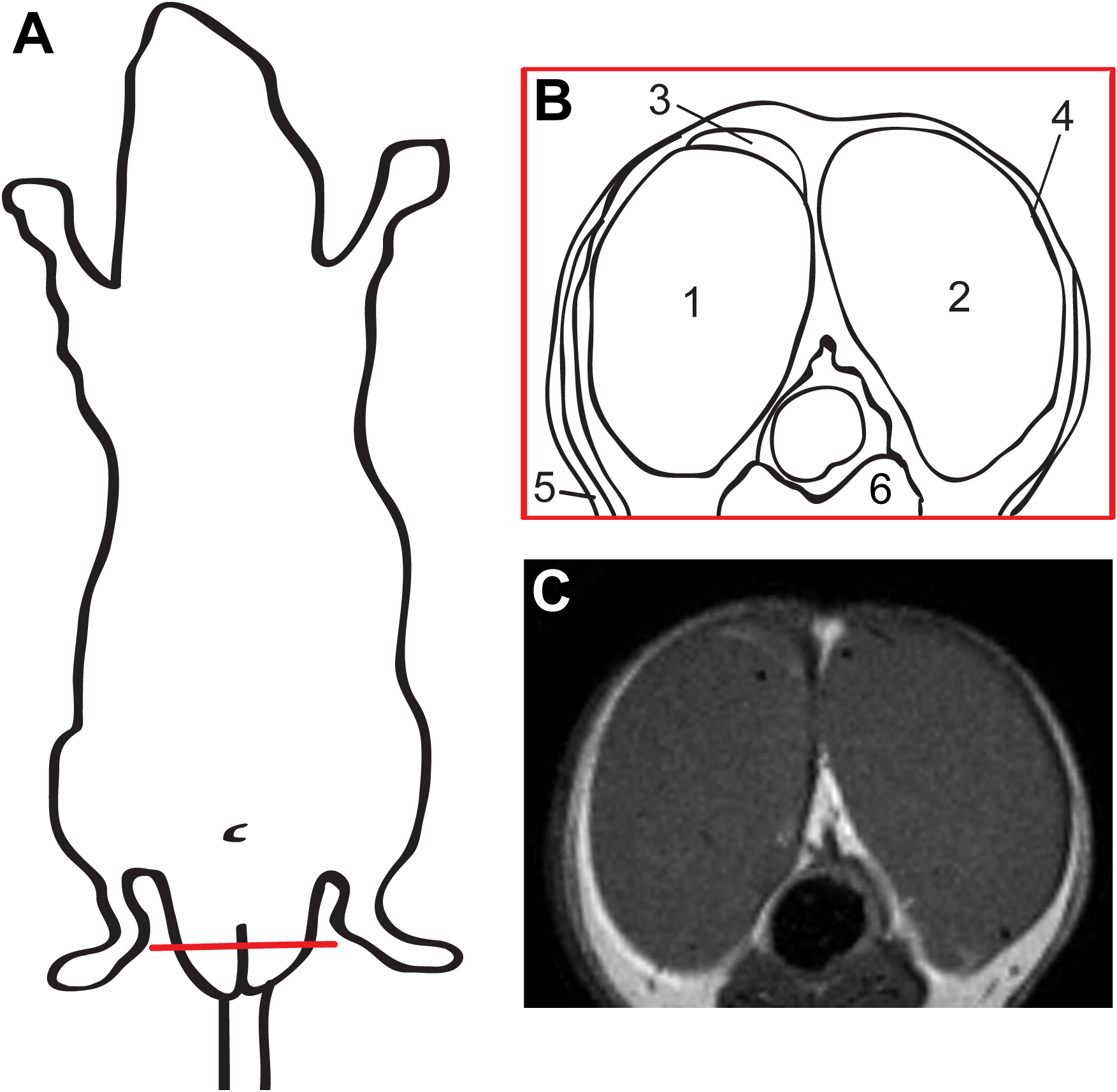

Supplement: Figure S1 — Labeled anatomical guide to interpreting rat testes MRI. (A): Cartoon outline of a rat in the supine position, as during MRI scanning. Red line through testes indicates the position of a representative image slice in Figure 1. (B): Cartoon depicting the cross-sectional (axial) view of the slice in (A). This image corresponds to the actual MRI slice shown in (C). Legend: 1 = right testis; 2 = left testis; 3 = epididymis; 4 = tunica albuginea; 5 = scrotum; 6 = tail. (C): Actual MRI image of testes from a sham-operated animal. (TIF) [file pone.0016456.s001.tif]

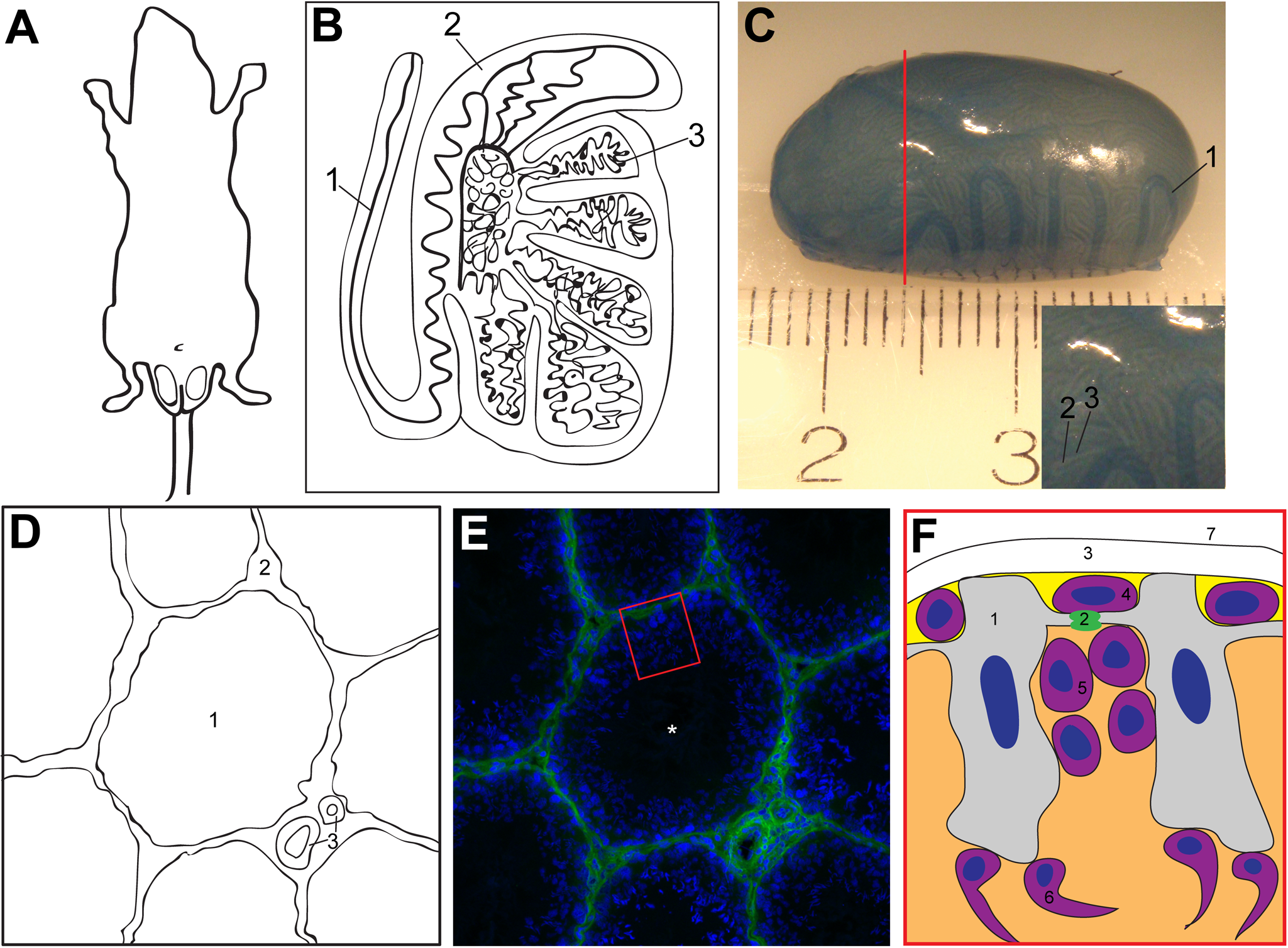

Supplement: Figure S2 — Anatomical guide to the testes and seminiferous tubules. (A): Cartoon outline of a rat illustrating the approximate size, shape, and location of testes. (B): Cartoon cross-section of a rat testis. Legend: 1 = collecting duct; 2 = epididymis; 3 = seminiferous tubules. (C): Photograph of a testis from a naïve rat that received intravascular Evans Blue (an albumin-binding dye). The blood vessels (1) on the testicular surface appear blue because they contain albumin-rich blood. Red line indicates plane of sectioning for histological studies. Inset shows the coiled seminiferous tubules (2), visible in contrast against the darker blue interstitial space (3). Ruler denotes length in cm. (D): Cartoon outline of a representative histological section of testis tissue. Legend: 1 = seminiferous tubule; 2 = interstitial space; 3 = blood vessels. (E): Actual immunofluorescent image of the seminiferous tubule depicted in (D). The nuclear stain DAPI is shown in blue. Asterisk indicates the lumen of a seminiferous tubule. Immunoreactivity for the blood-born protein immunoglobulin G is shown in green, to emphasize the distinct border between the interstitial space and the seminiferous tubules. Red box corresponds to (F). (F): Cartoon depicting cells of the seminiferous epithelium and the blood-testis barrier. Legend: 1 = Sertoli cell; 2 = tight junction (blood-testis barrier); 3 = basal lamina; 4 = spermatogonium; 5 = spermatocyte; 6 = spermatid; 7 = interstitial space. The blood-testis barrier separates the seminiferous tubule into two distinct compartments: basal (yellow) and adluminal (orange). (TIF) [file pone.0016456.s002.tif]
